# Integrated Meta-Analysis of Scalp Transcriptomics and Serum Proteomics Defines Alopecia Areata Subtypes and Core Disease Pathways

**DOI:** 10.3390/ijms26199662

**Published:** 2025-10-03

**Authors:** Li Xi, Elena Peeva, Yuji Yamaguchi, Zhan Ye, Craig L. Hyde, Emma Guttman-Yassky

**Affiliations:** 1 Pfizer Inc., Portland St., Cambridge, MA 02139, USA; 2 Pfizer Inc., Collegeville, PA 19426, USA; 3 Department of Dermatology, Icahn School of Medicine at Mount Sinai, New York, NY 10029, USA

**Keywords:** alopecia areata (AA), patchy alopecia (AAP), alopecia totalis and universalis (AT/AU), transcriptomics, proteomics, meta-analysis

## Abstract

Alopecia areata (AA) is a chronic autoimmune disorder characterized by non-scarring hair loss, with subtypes ranging from patchy alopecia (AAP) to alopecia totalis and universalis (AT/AU). The aim of this research is to investigate molecular features across AA severity by performing an integrated analysis of scalp transcriptomic datasets (GSE148346, GSE68801, GSE45512, GSE111061) and matched serum proteomic data from GSE148346. Differential expression analysis indicated that, relative to normal scalp, non-lesional AA tissue shows early immune activation—including Type 1 (*C-X-C motif chemokine ligand 9* (*CXCL9*), *CXCL10*, *CD8a molecule* (*CD8A*), *C-C motif chemokine ligand 5* (*CCL5*)) and Type 2 (*CCL13, CCL18*) signatures—together with reduced expression of hair-follicle structural genes (*keratin 32* *(KRT32)–35*, *homeobox C13* (*HOXC13*)) (FDR < 0.05, |fold change| > 1.5). Lesional AAP and AT/AU scalp showed stronger pro-inflammatory upregulation and greater loss of keratins and keratin-associated proteins (*KRT81, KRT83*, desmoglein 4 (*DSG4*), *KRTAP12/15*) compared with non-lesional scalp (FDR < 0.05, |fold change| > 1.5). Ferroptosis-associated genes (*cAMP responsive element binding protein 5* (*CREB5*), *solute carrier family 40 member 1* (*SLC40A1*), (lipocalin 2) *LCN2*, *SLC7A11*) and IRS (inner root sheath) differentiation genes (*KRT25, KRT27, KRT28, KRT71–KRT75, KRT81, KRT83, KRT85–86*, trichohyalin (*TCHH*)) were consistently repressed across subtypes, with the strongest reductions in AT/AU lesions versus AAP lesions, suggesting that oxidative-stress pathways and follicular structural integrity may contribute to subtype-specific pathology. Pathway analysis of lesional versus non-lesional scalp highlighted enrichment of IFN-α/γ, cytotoxic, and IL-15 signaling. Serum proteomic profiling, contrasting AA vs. healthy controls, corroborated scalp findings, revealing parallel alterations in immune-related proteins (CXCL9–CXCL10, CD163, interleukin-16 (IL16)) and structural markers (angiopoietin 1 (ANGPT1), decorin (DCN), chitinase-3-like protein 1 (CHI3L1)) across AA subtypes. Together, these data offer an integrated view of immune, oxidative, and structural changes in AA and found ferroptosis-related and IRS genes, along with immune signatures, as potential molecular indicators to support future studies on disease subtypes and therapeutic strategies.

## 1. Introduction

Alopecia areata (AA) is an autoimmune disease characterized by non-scarring hair loss, varying in extent from patchy alopecia (AAP) to more extensive forms such as alopecia totalis (AT) and universalis (AU). While immune-mediated attack on hair follicles is central to AA pathology, the molecular distinctions across subtypes remain inadequately understood [1,2,3]. Previous studies have highlighted roles for interferon signaling, CD8+ T-cell cytotoxicity, and keratinocyte stress responses [4,5,6]. The aim of this study is to perform comprehensive subtype-specific comparisons that integrate lesional and non-lesional scalp with serum protein data—an approach not previously undertaken.

Moreover, the potential involvement of ferroptosis-associated genes (e.g., *SLC7A11*, *glutathione peroxidase* (*GPX4*), *acyl-CoA synthetase long chain family member 4* (*ACSL4*) [7]) and inner root sheath (IRS) keratin genes (e.g., *KRT25, KRT27-28, KRT71–KRT75, KRT81, KRT83, KRT85-86*) [8,9] in the molecular landscape of AA has not been systematically investigated across disease severities. Whether these genes differ between lesional and non-lesional tissue remains an important unaddressed question.

To fill these gaps, we performed a meta-analysis across four publicly available scalp transcriptomic datasets (GSE148346, GSE688014, GSE45512, GSE111061) [3,4,5,6,10] and integrated the results with matched serum proteomic data from patients in the clinical trial GSE148346 [3]. Meta-analyzed transcriptomic profiles, followed by pathway enrichment analyses, enabled refined characterization of immune, oxidative, and structural programs across AA subtypes, expanding upon previously published transcriptomic characterizations of AA subtypes [5,10]. Importantly, incorporating serum proteomic profiles provided independent confirmation, revealing AA subtype-specific differences in key immune mediators compared to normal controls. Details of the datasets, including demographics, sample sizes, and sample types, are summarized in Supplementary Tables S1 and S2.

## 2. Results

### 2.1. Subclinical Immune Activation and Early Keratin Changes in Non-Lesional AAP Scalp

**Figure 1:**
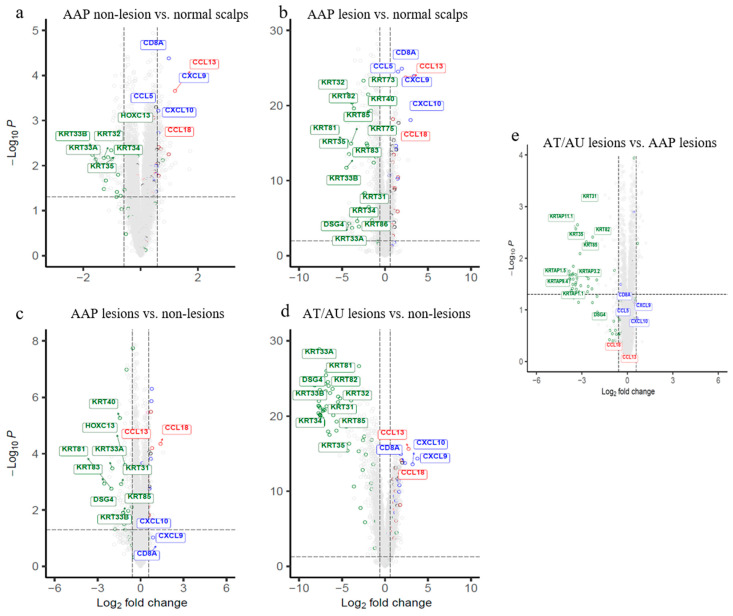
Volcano plots depicting differential gene expressions, and the heatmap exhibiting pathway dysfunction across AA subtypes and scalp regions. (**a**–**e**) Genes meeting FDR < 0.05 and |fold change| > 1.5 are shown for each comparison. Hair follicle genes (green), Type 1 (blue), and Type 2 (red) immunity genes are labeled. (**a**) Non-lesional scalp vs. normal shows early immune activation. (**b**) AAP lesions vs. normal reveals strong immune upregulation and keratin suppression. (**c**) AAP lesions vs. non-lesional highlight localized cytotoxicity and follicular loss. (**d**) AT/AU lesions vs. non-lesions shows widespread keratin repression with immune activation. (**e**) AT/AU lesions vs. AAP lesions exhibit greater hair shaft suppression with persistent immune signals. AAP = patchy-type AA; AT = alopecia totalis; AU = alopecia universalis; NL = non-lesional; NC = normal control; LS = lesional. The horizontal dotted line represents FDR = 0.05, and the vertical dotted lines represent an absolute fold change of 1.5.

This analysis delineates subtype-specific molecular profiles, highlighting a gradient of severity from non-lesional to lesional states. Non-lesional AAP scalp compared to normal controls identified distinct patterns of upregulated immune and metabolic genes as well as downregulated structural and regulatory genes. Non-lesional AAP scalp exhibited early immune activation, marked by elevated expression of Type 1 immunity genes (*CXCL9, CXCL10, CCL5, CD8A*, *killer cell lectin like receptor K1* (*KLRK1*)) and Type 2–linked chemokines (*CCL13, CCL3, CCL3L1*, *C-C motif chemokine ligand 3 like 3* (*CCL3L3*)), and innate receptors (*toll-like receptor 1* (*TLR1*), *C-type lectin like 1* (*CLECL1*)), along with downregulation of hair follicle–associated genes (*HOXC13,KRT33A/B,KRT34/35*), but remained molecularly more similar to normal scalp than to lesional areas. (Figure 1a, Supplemental Table S3).

### 2.2. Lesional AAP Scalp Shows Enhanced Immune Activation and Downregulation of Follicular Structural Genes

Lesional AAP scalp vs. normal controls exhibited strong immune activation with upregulation of T cell–associated genes (*CD3d molecule* (*CD3D*), *IL2 inducible T-cell kinase* (*ITK*), *LCK proto-oncogene, Src family tyrosine kinase* (*LCK*)), cytotoxic effectors (*perforin 1* (*PRF1*), *granzyme B* (*GZMB*), *GZMA, GZMK*), chemokines and immune mediators (*CCL18, CCL13, CCL5, CD2, CD28, CD1C, CD209*, *SLAM family member 8* (*SLAMF8*)), and antigen-presenting cell markers (*CD83, CD86*). In parallel, key follicular genes, including *HOXC13, DSG4*, multiple keratins (*KRT2, KRT33A/B, KRT38, KRT81, KRT83*), and keratin-associated proteins (*KRTAP1-3, KRTAP10-12, KRTAP4-2, KRTAP4-8, KRTAP9-9*), were markedly suppressed. Additionally, comparison to non-lesional scalp showed immune activation (*interleukin 12 receptor subunit beta 1* (*IL12RB1*), *integrin subunit alpha L* (*ITGAL*), *integrin subunit alpha M* (*ITGAM*), *phosphoinositide-3-kinase regulatory subunit 5* (*PIK3R5*), *cytohesin 1 interacting protein* (*CYTIP*), *sterile alpha motif domain containing 9 like* (*SAMD9L*)) and *matrix remodeling (matrix metallopeptidase 12* (*MMP12*), *collagen type VI alpha 5 chain* (*COL6A5*), *collagen type I alpha 2 chain* (*COL1A2*), *collagen type III alpha 1 chain* (*COL3A1*), *integrin binding sialoprotein* (IBSP), *ubiquitin D* (*UBD*)) (Figure 1b,c, Supplemental Table S4).

### 2.3. AT/AU Lesions Show Amplified Inflammation and Deeper Keratin Loss Compared to AAP Lesions

Lesional scalp from patients with alopecia totalis/universalis (AT/AU) revealed a distinct molecular signature associated with disease severity (Figure 1e, Supplemental Table S5). A large cluster of hair shaft keratins and keratin-associated proteins (KRTAPs), including *KRT81, KRT83, KRT86, KRT71, KRT74*, and *DSG4*, were significantly downregulated in AT/AU lesions. Additionally, extracellular matrix components (*cartilage oligomeric matrix protein* (*COMP*), *cellular communication network factor 2* (*CCN2*), *collagen type XI alpha 1 chain* (*COL11A1*), *hyaluronan and proteoglycan link protein 1* (*HAPLN1*)), *growth factors and developmental regulators (amphiregulin* (*AREG*), *bone morphogenetic protein 2* (*BMP2*), *HOXC13*, *forkhead box E1* (*FOXE1*), *CREB5*), and cell adhesion molecules (F11 receptor (*F11R*), *gap junction protein beta 6* (*GJB6)*, *epithelial membrane protein 1* (*EMP1*)) showed reduced expression in AT/AU compared to AAP lesions. Conversely, immune and inflammatory genes were upregulated in AT/AU lesions, with increased expression of *IL15*, *CXCR2*, *CXCL10*, *CXCL11*, *proteoglycan 2, bone marrow*(*PRG2*), and *matrix metallopeptidase 27* (*MMP27*). These changes were accompanied by enrichment of immune signaling pathway genes, including Janus kinase/signal transducer and activator of transcription (JAK/STAT) and interferon responses (Figure 1e, Supplemental Table S5).

### 2.4. Evaluation of Ferroptosis-Associated Genes and IRS (Inner Root Sheath) Keratin Genes

Analysis of the scalp transcriptome revealed a clear coherent downregulation of redox-related and structural programs across all contrasts examined—AAP lesional (LS) versus non-lesional (NL) scalp, AAP LS versus normal scalp, AT/AU LS versus normal scalp, and AT/AU LS versus AAP LS. The ferroptosis-associated genes *CREB5*, *SLC40A1*, *LCN2*, and *SLC7A11* were significantly decreased in each comparison (FDR < 0.05, |fold change| > 1.5), with the largest fold reductions observed in AT/AU LS relative to AAP LS, indicating progressive repression with disease severity. Genes involved in IRS (inner root sheath) differentiation showed a parallel pattern. IRS-specific keratins (*KRT25, KRT27, KRT28, KRT71, KRT72, KRT73, KRT74, KRT75, KRT81, KRT83, KRT85, KRT86*) were consistently downregulated across subtypes, and the IRS structural protein TCHH displayed marked suppression in AT/AU LS vs. AAP LS (logFC= −2.37, FDR < 0.05). Adhesion molecules *DSG3* and *desmocollin 3* (*DSC3*) were likewise reduced (logFC = −1.3). Other IRS/shaft-companion markers (*KRT32, KRT35, KRT36, KRT39*) followed the same trajectory, while *FXYD* domain containing ion transport regulator 1 (FXYD1) showed no significant change.

### 2.5. Pathway and Systemic Correlates Across Disease Stages

**Figure 2:**
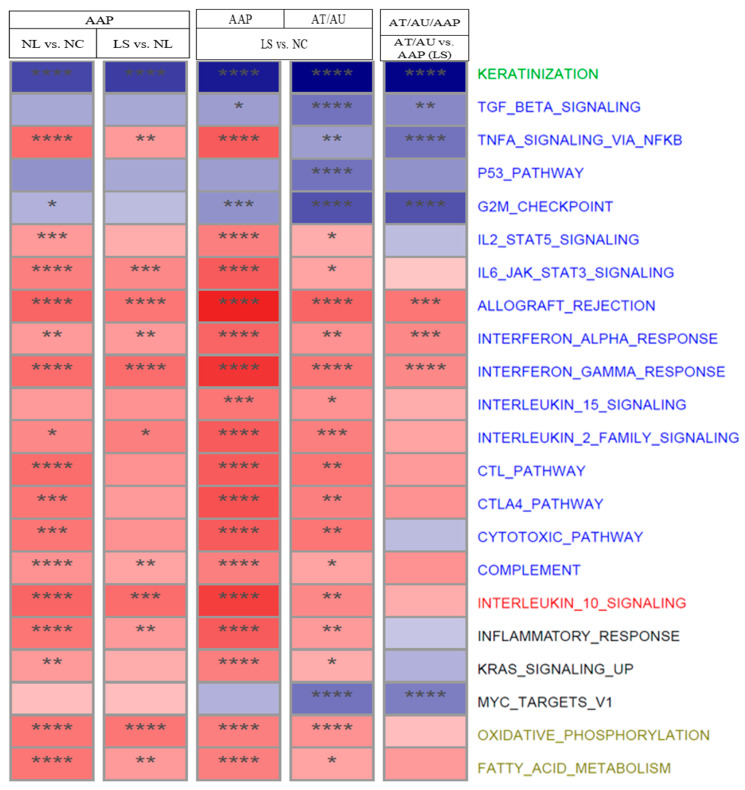
Pathway dysfunction across AA subtypes and scalp regions. The heatmap shows normalized enrichment scores (NES) for selected pathways across scalp comparisons: AAP NL vs. NC, LS vs. NL, LS vs. NC, and AT/AU LS vs. AAP LS. Red indicates activation, and blue indicates suppression in the listed comparisons. Asterisks denote significance (**** FDR < 0.001, *** FDR < 0.01, ** FDR < 0.05, and * FDR < 0.1). Pathways are color-coded by function: keratinization (green), immune (blue), regulatory (red), other (black) and metabolic (khaki).

Pathway-level enrichment analysis (Figure 2) revealed stage-specific modulation of immune pathways (IFN-α/γ and IL-6–JAK–STAT signaling) across disease severity. In contrast, the tumor necrosis factor alpha–signaling via nuclear factor kappa-light-chain-enhancer of activated B cells (TNFα–signaling via NFκB) pathway showed more pronounced suppression in AT/AU than in AAP. Similarly, pathways associated with cell cycle progression and metabolism—including tumor protein p53 (p53) signaling, MYC proto-oncogene (MYC) targets, and the G2/M checkpoint—were progressively downregulated, with the greatest suppression in AT/AU scalp. Notably, the keratinization pathway was downregulated in AAP and showed markedly greater suppression in AT/AU, consistent with impaired epithelial differentiation in advanced disease stages.

### 2.6. Concordant Serum Protein Changes

**Figure 3:**
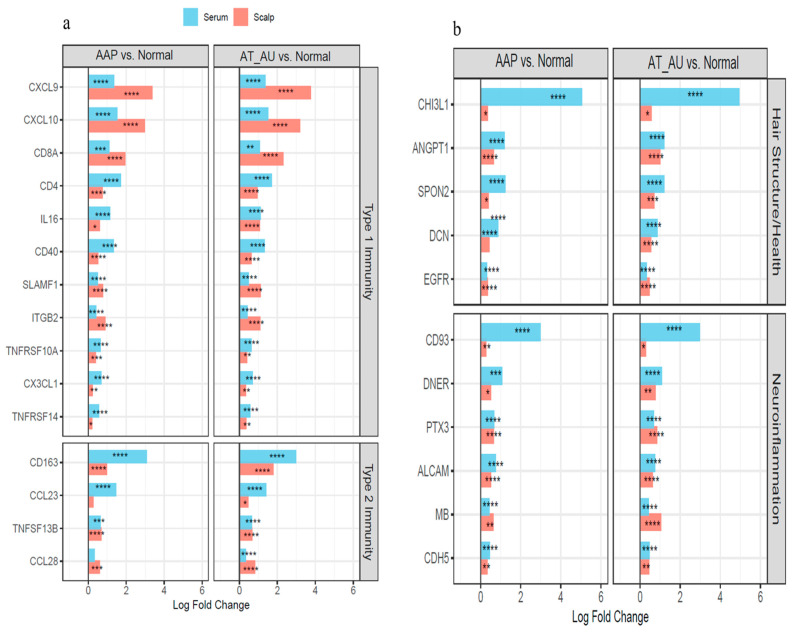
Coordinated immune activation and tissue remodeling in the scalp and serum of AA subtypes. (**a**) Type 1 and Type 2 immunity-related genes show parallel upregulation in both scalp and serum, reflecting local and systemic immune activation. (**b**) Hair structure and neuroinflammation-related genes are consistently elevated across tissues, indicating follicular disruption and inflammatory remodeling. Bars represent log fold changes in AAP and AT/AU vs. controls; red = scalp, blue = serum. **** FDR < 0.0001, *** FDR < 0.001, ** FDR < 0.01, and * FDR < 0.05.

Serum protein analysis revealed concordant changes with tissue-level alterations. Proteins including CX3CL1, CD4, CD40, ANGPT1, DCN, and CHI3L1 reflected scalp immune and structural gene signatures, indicating systemic immune activation across AA subtypes (Figure 3a,b).

## 3. Discussion

This meta-analysis of scalp transcriptomes delineates a molecular continuum in alopecia areata (AA), ranging from subclinical immune activation in patch-type alopecia (AAP) to extensive cytotoxic and structural pathology in severe forms such as alopecia totalis/universalis (AT/AU) [2,5,6,11]. Our findings highlight distinct molecular profiles between non-lesional and lesional scalps, with progressive intensification of immune signaling, keratin loss, and metabolic dysfunction across disease stages.

### 3.1. Non-Lesional AAP Scalp Vs. Normal Scalps

In non-lesional AAP scalp, early immune activation precedes clinical hair loss. Upregulation of Type 1 and Type 2 immunity genes including *CXCL9, CXCL10, CCL5, CD8A, KLRK1, CCL13, and CCL18*—suggests recruitment of cytotoxic and helper T cells even in unaffected regions, consistent with cytotoxic T-cell infiltration and IFN-γ pathway engagement in AA pathogenesis [5,6]. This immune priming was accompanied by relatively subtle suppression of follicular keratins and key structural regulators (*KRT32, KRT35, KRT33A, KRT33B, and HOXC13*), indicating early disruption of the follicular environment [2,3]. Previous studies have reported elevated serum levels of *CXCL9* and *CXCL10* in AA patients [12]. This adds to transcriptomic evidence of such immune activation in non-lesional scalp before visible hair loss. The metabolic upregulation (*fatty acid synthase* (*FASN*), *cholesterol 25-hydroxylase* (*CH25H*), *propionyl-CoA carboxylase beta subunit* (*PCCB*), *acyl-CoA synthetase short chain family member 2* (*ACSS2*)), supports the presence of subclinical immune activity and tissue stress [13,14]. These findings are consistent with previous reports of inflammatory infiltration in normal-appearing AA scalp [5,13]. Meanwhile, suppressed expression of follicular transcriptional regulators (*distal-less homeobox 3* (*DLX3*), *Msh homeobox 2* (*MSX2*), *Sp6 transcription factor* (*SP6*)) and barrier genes (*peptidyl arginine deiminase 1* (*PADI1*), *filaggrin antisense RNA 1* (*FLG-AS1*)) indicates early follicular dysregulation and keratinocyte differentiation impairment [15], highlighting targets in both Type 1 and 2 immunity pathways and metabolic regulation for early intervention [16].

### 3.2. Lesional AAP Vs. Normal Controls/Non-Lesional AAP

Lesional AAP scalp show a lesional signature dominated by T-cell activation, cytotoxicity, and antigen presentation, consistent with AA pathogenesis [1,2,3]. The strong downregulation of hair keratins (*KRT2, KRT33A/B, KRT38, KRT81, KRT83*) and KRTAPs indicates structural disruption and compromised follicular integrity, which are hallmarks of lesional AAP [5,6]. Transcription factors (*MSX2, CREB5, BNC2*) and adhesion molecules (*DSG4*, *plakophilin 1* (*PKP1*)) are also suppressed, suggesting a collapse of follicular maintenance programs [11]. In parallel, the upregulation of chemokines (*CCL5, CCL18*), cytotoxic markers (*PRF1, GZMB*), and Th1-associated genes (*CD28*, *Eomesodermin* (*EOMES*)) reflect a robust immune response, consistent with the central role of cytotoxic T cells and NK cells in alopecia pathogenesis [1,2,14]. Elevated matrix remodeling genes (*MMP12, COL6A5, COL3A1*) highlight active extracellular matrix turnover, which may contribute to perifollicular fibrosis and lesion persistence [16].

### 3.3. Lesional AT/AU Vs. AAP

In AT/AU Vs. AAP, the results demonstrate that AT/AU lesions exhibit a dual molecular pattern, with downregulation of structural genes critical for follicular integrity and upregulation of inflammatory and immune pathways. The loss of KRT and KRTAP gene expression (*KRT81, KRT83, KRT86, DSG4*) and suppression of key extracellular matrix components (*COMP, CCN2, COL11A1*) reflect extensive hair shaft fragility and follicular miniaturization, in line with previous findings of structural protein loss in severe alopecia areata [5,6]. The increased expression of immune and inflammatory mediators (*IL15, CXCR2, CXCL10*, *S100 calcium binding protein A2* (*S100A2*), *Kallikrein related peptidase 6* (*KLK6*), *MMP27*) highlights a heightened immune response, consistent with the involvement of cytotoxic T cells and activation of JAK/STAT and interferon signaling pathways [11,14]. The concurrent repression of regenerative markers (*AREG, FOXE1, HOXC13, BMP2*) suggests that AT/AU lesions represent a more advanced, less reversible disease state compared to AAP, where structural programs remain partially active [1,2,17,18].

### 3.4. Ferroptosis-Associated Genes and IRS (Inner Root Sheath) Keratin Genes

The current work extends to identify differential signals in *SLC40A1*, *LCN2*, *CREB5*, and *SLC7A11* as ferroptosis-related biomarkers in alopecia areata [7]. Their consistent suppression across AAP and AT/AU lesions, with a graded decline from patchy AA to advanced AT/AU, suggests that vulnerability to ferroptosis and/or oxidative injury is an intrinsic component of follicular pathology in AA [7,19]. This redox imbalance may synergize with immune-mediated injury to perpetuate hair follicle damage [19,20]. Simultaneous repression of IRS keratins, trichohyalin, and desmosomal components (*DSG3*, *DSC3*) underscores a convergent defect in IRS integrity. Loss of these structural elements likely compromise hair-shaft anchorage and differentiation, providing a mechanistic link between oxidative stress, epithelial support failure, and hair-cycle arrest [8,9,21].

The integration of ferroptotic stress markers with IRS-related genes therefore defines a progressive follicular vulnerability program, highlighting potential therapeutic avenues aimed at restoring redox balance and/or protecting IRS differentiation alongside immune modulation [8,9,21].

Of note, because bulk transcriptomics averages signals across follicular compartments, the specific hair-cell layers contributing to immune and structural changes cannot be determined. Single-cell RNA-seq and spatial transcriptomic approaches will be needed to map immune and structural signatures to specific follicular cell types.

### 3.5. Pathway Enrichment Analysis Across AA Subtypes

Pathway analysis supported these findings, revealing progressive enrichment of immune-related signaling across disease stages. Non-lesional AAP scalp showed early activation of interferon-α/γ pathways, while AAP lesions exhibited increased cytotoxic T cell signaling, NF-κB activation, and suppression of keratinization and cell cycle genes. Oxidative phosphorylation and fatty acid metabolism were already enriched in AAP and became more pronounced in AT/AU, reflecting sustained metabolic impairment [6]. Persistent upregulation of interferon, cytotoxic T cell, and IL-6/JAK/STAT3 pathways highlights chronic immune-mediated injury and a transition to a transcriptionally advanced disease state in AT/AU.

Beyond immune priming through JAK/STAT, lipid peroxidation and iron-dependent cell death pathways may contribute to early follicular injury [19,20]. Recent reports implicating ferroptosis-related genes (*SLC7A11*, *GPX4*, *ACSL4*) in inflammatory skin disorders might suggest that oxidative stress and ferroptosis may potentially interact with immune pathways in AA, representing promising targets for future mechanistic and translational studies [7,19].

Findings for ferroptosis-related transcripts and IRS keratins suggest additional processes—oxidative stress and follicular structural integrity—may shape subtype-specific pathology [7,19,20]. These pathway genes merit functional evaluation and could potentially inform biomarkers or therapeutic development aimed at preserving follicular viability [8,9,20,21].

### 3.6. Proteomic Profiles Across AA Subtypes

Integration of serum proteomic data further supported systemic involvement. Scalp upregulation of *CXCL9*, *CXCL10*, and *ITGB2* reflected localized CD8^+^ T cell–driven inflammation, while increased serum levels of CX3CL1, CD4, and CD40 indicated systemic immune activation. Proteins such as ANGPT1, CHI3L1, and DCN, involved in vascular remodeling and extracellular matrix dynamics, were elevated in serum and mirrored keratin and matrix suppression observed in scalp tissue. These concordant changes suggest that AA progression involves both localized immune infiltration and broader systemic immune dysregulation [4,22].

Together, these findings are consistent with a model in which early immune activation and keratin disruption in AAP non-lesional scalp represent a pre-lesional state that can evolve into full immune-mediated follicular collapse. Type 1 and Type 2 immune responses play central roles in driving this progression. AT/AU represents a more advanced molecular stage of disease with persistent cytotoxicity, structural breakdown, and metabolic dysfunction. Therapeutically, these results highlight the importance of early intervention in AAP to prevent progression to irreversible follicular loss in AT/AU. Targeting interferon signaling, cytotoxic T cell pathways, and keratin preservation may offer strategies to halt or reverse AA progression. These signatures could be refined and validated in studies correlating clinical outcomes with molecular profiles. Mechanistic (in vitro/in vivo) studies will be needed for functional validation of the molecular signature reported in this study.

## 4. Materials and Methods

### 4.1. Transcriptomic Datasets

Four scalp transcriptomic datasets (GSE148346, GSE68801, GSE45512, GSE111061,) were analyzed, including lesional, non-lesional, and normal scalp samples from alopecia areata (AA) patients and controls. GSE148346 also includes matched serum proteomic data of patients from the clinical trial GSE148346.

### 4.2. Serum Proteomics

Baseline serum from GSE148346 was profiled using Olink’s proximity extension assay (INF, CVD II/III, NEU panels) (Waltham, MA, USA) [23]. Differential protein analyses were performed using a linear mixed model.

### 4.3. Data Processing and Differential Expression Analysis

All datasets used the Affymetrix (Waltham, MA, USA) HGU133 Plus 2.0 platform. Raw CEL files were normalized using R package GCRMA, version 2.80.0 [24], and differential expression analysis was performed using R package LIMMA, version 3.64.3 [25], with FDR < 0.05 [26] and |fold change| > 1.5 as significance thresholds. The Benjamini–Hochberg procedure was used to adjust *p* values for multiple hypotheses by controlling the false discovery rate (FDR) based on contrasts from each study.

### 4.4. Meta-Analysis

To enhance statistical power and identify robust molecular signatures, we performed a meta-analysis integrating multiple transcriptomic datasets (GSE148346, GSE68801, GSE45512, GSE111061). An inverse-variance weighted meta-analysis [27] was used to combine effect sizes and SEs across datasets using random effects model, allowing to account for heterogeneity between studies while identifying consistent patterns of differential expressions. Genes showing consistent direction of effect and low heterogeneity—quantified by Tau-squared statistics calculated using the DerSimonian and Laird (DL) method based on Q statistics—were prioritized. Genes with Tau-squared values close to zero were considered to have minimal heterogeneity across studies. Although residual heterogeneity was evaluated using the Q statistic and Tau-squared estimates, and while the random-effects model minimized study-specific influence, some variation between datasets might persist and should be considered when interpreting the result.

### 4.5. Pathway Enrichment

Ranked LIMMA t-statistics were analyzed using fgsea (fast gene set enrichment analysis) [28] with HALLMARK, REACTOME, BIOCARTA, and KEGG gene sets. Pathways with NES (normalized enrichment score) > 2 and FDR ≤ 0.05 were considered significant.

## 5. Conclusions

This study integrates transcriptomic meta-analysis and serum proteomics to distinguish molecular programs across alopecia areata subtypes. We identified robust immune signatures differentiating AAP, AT, and AU, comparing lesional to normal, and/or non-lesional, non-lesional AAP vs. normal scalps, or lesional AT/AU vs. lesional AAP, and uncovered additional differential signals from ferroptosis-associated genes and IRS keratin genes, implicating oxidative stress and follicular structural integrity in disease heterogeneity. Key immune markers showed consistent differences in tissue and serum when comparing AA subtypes with normal controls, underscoring their utility as potential biomarkers. These findings refine molecular characterizations of AA subtypes and highlight future research interest, including functional validation of ferroptosis and IRS pathways in AA disease, application of single-cell and spatial omics approaches, and development of therapeutic strategies to preserve follicular viability and support hair regrowth.

## Data Availability

The data presented in this study are openly available in [GEO, reference GSE148346, GSE68801, GSE45512, GSE111061.

## Supplementary Materials

The following supporting information can be downloaded at: https://www.mdpi.com/article/10.3390/ijms26199662/s1.
