# Supplementary material for: Integrated Meta-Analysis of Scalp Transcriptomics and Serum Proteomics Defines Alopecia Areata Subtypes and Core Disease Pathways
Source: Int J Mol Sci. 2025 Oct 3;26(19):9662. doi: 10.3390/ijms26199662 (PMC12525155; doi:10.3390/ijms26199662)
Supplement: Supplementary file 1 [file ijms-26-09662-s001.zip › ijms-3826431-supplementary.pdf]

Supplementary Information

Integrated Meta-Analysis of Scalp Transcriptomics and Serum Proteomics Defines Alopecia Areata Subtypes and Core Disease Pathways

Li Xi[1], Elena Peeva[1*], Yuji Yamaguchi[2], Zhan Ye[1], Craig L Hyde[1], Emma Guttman-Yassky[3]

Affiliations

1. Pfizer Inc, Cambridge, MA, USA;
2. Pfizer Inc, Collegeville, PA, USA;
3. Department of Dermatology, Icahn School of Medicine at Mount Sinai, New York, NY, USA

*Correspondence: elena.peeva@pfizer.com
1 Portland St.
Cambridge, MA, 02139
USA

This document provides additional data, figures, and methods supporting the main manuscript. Each supplementary item is described below with legends and cross-references to the main text.

Supplementary Tables

• Supplementary Table S1: [Description of data sets used in these analyses]

Study – GEO	PMID	N	Age	Sex F/M	Race	Baseline SALT score	Type (AAP – AT/AU)	Duration
GSE148346	34863853	12 (PBO)<br>18 (Ritlcitinib)<br>16 (Brepocitinib)	42.0±12.5 (mean±SD)	33/13		87.4±18.0 (mean±SD)	33 – 13	2.4 (1.1 – 3.9) Median (IQR)
GSE68801	27699252	66	37 (19-65) Median (range)	31/35	N/A		52 - 14	5 (0.5 – 43) median (range)
GSE45512	25129481	10	18-75 (range)	N/A	N/A	30 to 95% hair loss due to alopecia areata (AA) as measured by SALT score	10	Hair loss duration of at least 3 months
GSE111061	29452121	12	N/A	8/4	5 Caucasian<br>4 African<br>3 Hispanic		5 - 7	

• Supplementary Table S2: [Data sets with sample sizes by AA disease subtypes]

Studies - GEO	Sample types (Sample size)	Publication
GSE148346	Tissue:<br>AAP<br>• LS (16)<br>• NL (18)<br>AT/AU<br>• LS (26)<br>Serum samples (113)	Guttman-Yassky, E., et al. (2022). Ritlecitinib and brepocitinib demonstrate significant improvement in scalp alopecia areata biomarkers. *Journal of Allergy and Clinical Immunology, 149*(4), 1324-1332.
GSE68801	Tissue:<br>AAP<br>• LS (28)<br>• NL (26)<br>AT/AU<br>• LS (32)<br>NC (36)	Jabbari A, et al (2016). Molecular signatures define alopecia areata subtypes and transcriptional biomarkers. EBioMedicine. 7:240-7.
GSE45512	Tissue:<br>AAP<br>• LS (5)<br>NC (5)	Xing L, et al (2014). Alopecia areata is driven by cytotoxic T lymphocytes and is reversed by JAK inhibition. Nat Med. 20(9):1043-9.
GSE111061	Tissue:<br>AAP<br>• LS (5)<br>NC (6)	Jabbari A, et al (2018). An Open-Label Pilot Study to Evaluate the Efficacy of Tofacitinib in Moderate to Severe Patch-Type Alopecia Areata, Totalis, and Universalis. J Invest Dermatol. 138(7):1539-1545.

AA: alopecia areata, AAP: alopecia areata patchy, AT: alopecia totalis, AU: alopecia universalis

LS: lesional, NL: non-lesional, NC: normalSupplementary Table S3: [Comparison analysis AAP NL vs. NC]

Genes filtered by FDR < 0.2 & |Fold change| > 1.5

SYMBOL	GENENAME	logFC	CI.L	CI.R	t	P.Value	adj.P.Val	contrast

SYCE1L	synaptonemal complex central element protein 1 like	-0.59495	-0.84653	-0.34337	-4.72581	1.33E-05	0.121165	aap.nl.vs.nc
DLX3	distal-less homeobox 3	-0.95809	-1.35436	-0.56183	-4.83163	9.04E-06	0.121165	aap.nl.vs.nc
CD8A	CD8a molecule	0.985658	0.538687	1.43263	4.406766	4.16E-05	0.128462	aap.nl.vs.nc
SSBP1	single stranded DNA binding protein 1	0.720962	0.384025	1.057899	4.276001	6.58E-05	0.128462	aap.nl.vs.nc
UHRF1BP1L	UHRF1 binding protein 1 like	0.653818	0.35086	0.956775	4.312696	5.79E-05	0.128462	aap.nl.vs.nc
RMND5A	required for meiotic nuclear division 5 homolog A	-0.66011	-0.94731	-0.37292	-4.59317	2.15E-05	0.128462	aap.nl.vs.nc
PCCB	propionyl-CoA carboxylase subunit beta	0.662038	0.348106	0.97597	4.214257	8.15E-05	0.138303	aap.nl.vs.nc
HSPA14	heat shock protein family A (Hsp70) member 14	-0.65093	-0.96244	-0.33943	-4.17585	9.30E-05	0.138303	aap.nl.vs.nc
FLG-AS1	FLG antisense RNA 1	-0.87669	-1.29872	-0.45466	-4.15126	0.000101	0.138303	aap.nl.vs.nc
CXCL9	C-X-C motif chemokine ligand 9	1.635477	0.837712	2.433242	4.096786	0.000122	0.141777	aap.nl.vs.nc
TLR1	toll like receptor 1	0.67128	0.335274	1.007287	3.992364	0.000174	0.151808	aap.nl.vs.nc
PPP1R2	protein phosphatase 1 regulatory inhibitor subunit 2	-0.92398	-1.38583	-0.46212	-3.99789	0.00017	0.151808	aap.nl.vs.nc
CALCB	calcitonin related polypeptide beta	-0.65631	-0.98583	-0.3268	-3.98021	0.000181	0.154418	aap.nl.vs.nc
CCL13	C-C motif chemokine ligand 13	1.201083	0.589105	1.813062	3.922026	0.00022	0.171331	aap.nl.vs.nc
CCL3	C-C motif chemokine ligand 3	0.602246	0.293982	0.91051	3.904136	0.000233	0.1716	aap.nl.vs.nc
CCL3L1	C-C motif chemokine ligand 3 like 1	0.602246	0.293982	0.91051	3.904136	0.000233	0.1716	aap.nl.vs.nc
CCL3L3	C-C motif chemokine ligand 3 like 3	0.602246	0.293982	0.91051	3.904136	0.000233	0.1716	aap.nl.vs.nc
ATF7IP2	activating transcription factor 7 interacting protein 2	0.745552	0.35719	1.133913	3.836325	0.000292	0.177074	aap.nl.vs.nc
ACSS3	acyl-CoA synthetase short chain family member 3	0.626271	0.300733	0.951808	3.844454	0.000284	0.177074	aap.nl.vs.nc
THRAP3	thyroid hormone receptor associated protein 3	-0.82943	-1.26439	-0.39446	-3.81064	0.000317	0.178896	aap.nl.vs.nc
KLRK1	killer cell lectin like receptor K1	0.701424	0.329347	1.073501	3.767226	0.000366	0.18351	aap.nl.vs.nc
KLRC4-KLRK1	KLRC4-KLRK1 readthrough	0.701424	0.329347	1.073501	3.767226	0.000366	0.18351	aap.nl.vs.nc
CLECL1	C-type lectin like 1	0.66163	0.310955	1.012305	3.77037	0.000362	0.18351	aap.nl.vs.nc
SLC40A1	solute carrier family 40 member 1	-0.87572	-1.33918	-0.41227	-3.776	0.000356	0.18351	aap.nl.vs.nc
LDHD	lactate dehydrogenase D	1.005441	0.46812	1.542763	3.739349	0.000401	0.183687	aap.nl.vs.nc
ROS1	ROS proto-oncogene 1, receptor tyrosine kinase	0.994375	0.453956	1.534793	3.676999	0.00049	0.183687	aap.nl.vs.nc
C1QB	complement C1q B chain	0.685949	0.316875	1.055023	3.714087	0.000435	0.183687	aap.nl.vs.nc
HYKK	hydroxylysine kinase	0.620578	0.289235	0.951922	3.742757	0.000396	0.183687	aap.nl.vs.nc
ACAA2	acetyl-CoA acyltransferase 2	0.599628	0.272209	0.927047	3.659753	0.000518	0.183687	aap.nl.vs.nc
MREG	melanoregulin	-0.7075	-1.09038	-0.32462	-3.69266	0.000466	0.183687	aap.nl.vs.nc
TCP11L2	t-complex 11 like 2	-0.75775	-1.17189	-0.34361	-3.65635	0.000524	0.183687	aap.nl.vs.nc
CXCL10	C-X-C motif chemokine ligand 10	1.34576	0.604233	2.087286	3.626724	0.000576	0.187715	aap.nl.vs.nc
CCL5	C-C motif chemokine ligand 5	0.619707	0.276766	0.962649	3.611103	0.000605	0.187715	aap.nl.vs.nc
METTL7B	methyltransferase like 7B	0.87512	0.389187	1.361053	3.598859	0.00063	0.188925	aap.nl.vs.nc
PADI1	peptidyl arginine deiminase 1	-0.92639	-1.44169	-0.41109	-3.59258	0.000642	0.188925	aap.nl.vs.nc
GPR171	G protein-coupled receptor 171	0.757624	0.329952	1.185297	3.540109	0.000758	0.191303	aap.nl.vs.nc
FASN	fatty acid synthase	0.731439	0.315127	1.147752	3.511011	0.000831	0.191303	aap.nl.vs.nc
CH25H	cholesterol 25-hydroxylase	0.693416	0.302278	1.084554	3.542724	0.000752	0.191303	aap.nl.vs.nc
ACSS2	acyl-CoA synthetase short chain family member 2	0.630519	0.270535	0.990503	3.500165	0.00086	0.191303	aap.nl.vs.nc
ASPH	aspartate beta-hydroxylase	0.602462	0.259697	0.945227	3.512426	0.000827	0.191303	aap.nl.vs.nc
RPH3AL	rabphilin 3A like (without C2 domains)	0.597535	0.259739	0.935331	3.534946	0.000771	0.191303	aap.nl.vs.nc
IL10RA	interleukin 10 receptor subunit alpha	0.589467	0.252755	0.926178	3.498441	0.000865	0.191303	aap.nl.vs.nc

CAMSAP1	calmodulin regulated spectrin associated protein 1	-0.58652	-0.91602	-0.25702	-3.55713	0.000719	0.191303	aap.nl.vs.nc
SMAD7	SMAD family member 7	-0.59766	-0.93216	-0.26317	-3.57059	0.000689	0.191303	aap.nl.vs.nc
GAPDHP62	glyceraldehyde 3 phosphate dehydrogenase pseudogene 62	-0.65132	-1.01595	-0.28668	-3.56952	0.000691	0.191303	aap.nl.vs.nc
ANKRD46	ankyrin repeat domain 46	-0.65132	-1.01595	-0.28668	-3.56952	0.000691	0.191303	aap.nl.vs.nc
MAF	MAF bZIP transcription factor	-0.68399	-1.0771	-0.29088	-3.47704	0.000924	0.191303	aap.nl.vs.nc
SP6	Sp6 transcription factor	-0.87611	-1.37198	-0.38024	-3.5307	0.000781	0.191303	aap.nl.vs.nc
MYCN	MYCN proto-oncogene, bHLH transcription factor	-0.94149	-1.47864	-0.40433	-3.50259	0.000853	0.191303	aap.nl.vs.nc
MSX2	msh homeobox 2	-0.96844	-1.52899	-0.40789	-3.45247	0.000998	0.193305	aap.nl.vs.nc
HGD	homogentisate 1,2-dioxygenase	0.69174	0.289625	1.093856	3.437684	0.001045	0.195702	aap.nl.vs.nc
FYB1	FYN binding protein 1	0.667842	0.277842	1.057843	3.422018	0.001097	0.195702	aap.nl.vs.nc
GMCL2	germ cell-less 2, spermatogenesis associated	-0.63497	-1.0047	-0.26525	-3.43202	0.001064	0.195702	aap.nl.vs.nc
GMCL1	germ cell-less 1, spermatogenesis associated	-0.63497	-1.0047	-0.26525	-3.43202	0.001064	0.195702	aap.nl.vs.nc
KIF16B	kinesin family member 16B	0.613356	0.253324	0.973388	3.404434	0.001158	0.198703	aap.nl.vs.nc
SLA	Src like adaptor	0.607328	0.250804	0.963853	3.404147	0.001159	0.198703	aap.nl.vs.nc

Supplementary Table S4: [Comparison analysis LS AAP vs. NL AAP]

Genes filtered by FDR < 0.05 & |Fold change| > 1.5

	Down in lesional AAP vs non-lesional AAP								Up in lesional AAP vs non-lesional AAP				
Gene	logFC	p.value	adj.P		Gene	logFC	p.value	adj.P		Gene	logFC	p.value	adj.P
DSG4	-2.598	4.02E-03	4.99E-02		NELL2	-0.845	1.10E-04	5.58E-03		MIAT	0.587	3.35E-05	2.67E-03
KRT83	-2.534	1.15E-03	2.40E-02		FETUB	-0.840	9.26E-08	7.10E-05		GZMA	0.588	9.48E-04	2.11E-02
KRTAP1-3	-2.161	6.72E-04	1.72E-02		PPP1R2	-0.838	2.01E-07	1.06E-04		SAMD9L	0.589	1.01E-05	1.26E-03
GPRC5D	-2.116	5.21E-05	3.55E-03		SLC5A9	-0.828	2.43E-03	3.73E-02		CYTIP	0.589	1.80E-03	3.08E-02
CHAC1	-2.062	4.02E-04	1.23E-02		LSMEM1	-0.798	2.34E-04	8.81E-03		PIK3R5	0.596	1.64E-04	7.14E-03
KRT81	-2.037	1.75E-03	3.03E-02		ZAR1	-0.785	3.04E-04	1.02E-02		CD3D	0.598	8.45E-05	4.77E-03
LY6G6D	-2.027	1.28E-04	6.09E-03		PKP1	-0.779	4.27E-04	1.27E-02		CD209	0.603	7.69E-05	4.56E-03
LY6G6F	-2.027	1.28E-04	6.09E-03		BNC2	-0.739	1.63E-03	2.92E-02		CD28	0.605	1.43E-03	2.73E-02
KRT33A	-1.973	3.32E-04	1.08E-02		FGG	-0.720	1.29E-06	3.50E-04		CD28	0.605	1.43E-03	2.73E-02
KRTAP10-12	-1.603	6.97E-05	4.23E-03		DACH1	-0.714	2.24E-05	2.03E-03		IBSP	0.609	1.68E-04	7.22E-03
KRTAP10-7	-1.603	6.97E-05	4.23E-03		IL1F10	-0.714	3.56E-03	4.78E-02		ITGAM	0.610	3.36E-06	6.46E-04
KRTAP4-2	-1.560	3.77E-03	4.87E-02		SEC14L2	-0.692	1.66E-07	9.43E-05		HLA-DQB1	0.623	2.28E-03	3.59E-02
KRTAP9-9	-1.550	3.70E-03	4.85E-02		MSX2	-0.680	1.56E-03	2.85E-02		GZMB	0.625	1.74E-03	3.03E-02
COMP	-1.517	1.93E-04	7.94E-03		MAP7D2	-0.674	1.69E-04	7.24E-03		ITGAL	0.626	1.32E-04	6.21E-03
FGF5	-1.500	2.82E-03	4.12E-02		EGF	-0.667	4.00E-07	1.60E-04		EOMES	0.630	8.07E-05	4.67E-03
CST1	-1.489	1.77E-06	4.35E-04		HSPA14	-0.655	1.02E-07	7.28E-05		IKZF1	0.645	5.34E-06	8.31E-04
PIRT	-1.459	5.09E-10	3.88E-06		CREB5	-0.647	7.56E-07	2.40E-04		GZMK	0.652	6.18E-04	1.64E-02
KRT40	-1.444	5.42E-06	8.33E-04		MREG	-0.643	7.17E-06	1.03E-03		COL3A1	0.661	1.16E-04	5.79E-03
KRTAP4-8	-1.412	2.09E-03	3.39E-02		PPARD	-0.636	9.36E-04	2.11E-02		SLAMF8	0.678	3.17E-04	1.06E-02
KRT31	-1.356	1.20E-03	2.47E-02		TMEM163	-0.633	5.25E-07	1.78E-04		TRG-AS1	0.679	3.51E-05	2.73E-03
RNF182	-1.266	1.61E-05	1.68E-03		SHF	-0.627	1.07E-09	5.42E-06		THEMIS	0.681	1.93E-05	1.83E-03
SLC7A11	-1.247	1.15E-03	2.39E-02		VSNL1	-0.623	1.25E-03	2.52E-02		CD2	0.697	9.85E-05	5.26E-03
CST4	-1.215	2.83E-04	9.81E-03		BMP2	-0.618	1.07E-04	5.49E-03		UBD	0.712	9.72E-04	2.15E-02
DNAJC6	-1.170	2.01E-05	1.90E-03		KLRK1-AS1	-0.613	8.49E-06	1.14E-03		PRF1	0.714	1.51E-04	6.83E-03
CTNND2	-1.156	1.65E-04	7.14E-03		ELF5	-0.608	1.49E-06	3.86E-04		CD1C	0.719	3.25E-06	6.40E-04
SERPINA1	-1.144	2.94E-05	2.45E-03		RASSF9	-0.608	7.37E-05	4.44E-03		CCL5	0.757	1.36E-06	3.64E-04
TNIK	-1.127	4.22E-05	3.09E-03		BEST3	-0.607	9.51E-05	5.19E-03		CCL5	0.757	1.36E-06	3.64E-04
GJA3	-1.125	2.67E-03	3.98E-02		H2BC11	-0.606	9.91E-05	5.26E-03		IL12RB1	0.785	4.97E-07	1.74E-04
HOXC13	-1.091	2.54E-04	9.17E-03		H2BC8	-0.606	9.91E-05	5.26E-03		COL1A2	0.786	2.53E-10	3.86E-06
ALDOB	-1.084	8.50E-06	1.14E-03		UBE2D1	-0.605	7.78E-04	1.87E-02		COL6A5	0.795	1.64E-04	7.14E-03
SYTL5	-1.045	5.35E-04	1.48E-02		PPP2R1B	-0.601	5.03E-07	1.74E-04		CCL13	0.803	6.33E-05	3.99E-03
PSORS1C2	-1.045	1.21E-04	5.96E-03		NELL1	-0.593	2.44E-03	3.73E-02		CCL18	1.393	4.47E-05	3.20E-03
BAMBI	-1.020	1.00E-03	2.21E-02		ARL15	-0.590	2.23E-04	8.55E-03		MMP12	1.576	9.05E-05	4.96E-03
KLK12	-1.015	6.64E-04	1.71E-02		PDZRN3	-0.586	2.48E-03	3.77E-02					
KRT16	-0.976	1.05E-07	7.28E-05		TENM2	-0.582	3.50E-03	4.72E-02					
TRIM9	-0.971	1.46E-03	2.76E-02		RMND5A	-0.582	2.79E-03	4.08E-02					
SHISA2	-0.969	1.45E-07	8.93E-05		KALRN	-0.859	8.04E-04	1.90E-02					
CYRIA	-0.959	3.73E-06	6.85E-04		FZD3	-0.845	7.45E-04	1.81E-02					

Supplementary Table S5: [Comparison analysis LS AT/AU vs. LS AAP]

Genes filtered by FDR < 0.05 & |Fold change| > 1.5

	Down in lesional AT/AU vs. lesional AAP									Up in lesional AT/AU vs. lesional AAP			
Gene	logFC	P.Value	FDR		Gene	logFC	P.Value	FDR		Gene	logFC	P.Value	FDR
KRTAP4-4	-5.557	8.64E-10	7.08E-06		ARSF	-0.941	3.09E-05	7.36E-03		ME3	0.581	4.16E-04	3.50E-02
KRTAP9-4	-5.493	3.44E-10	6.54E-06		HOMER1	-0.919	9.43E-05	1.50E-02		MAN1C1	0.582	2.85E-04	2.88E-02
KRTAP4-6	-5.479	4.20E-09	1.01E-05		BNC2	-0.918	6.03E-06	2.50E-03		FMOD	0.586	2.37E-04	2.56E-02
KRTAP2-3	-5.457	3.59E-10	6.54E-06		PRRG4	-0.914	2.33E-04	2.54E-02		CHDH	0.591	3.25E-04	3.06E-02
KRTAP4-12	-5.445	2.07E-09	8.70E-06		KRT73	-0.912	1.92E-04	2.28E-02		IL15	0.595	3.70E-04	3.26E-02
KRTAP1-5	-5.428	1.61E-09	7.92E-06		SLC1A6	-0.892	1.40E-04	1.94E-02		RNF150	0.597	6.75E-04	4.57E-02
KRTAP4-1	-5.411	2.87E-09	8.71E-06		DACH1	-0.874	7.44E-04	4.89E-02		LINC00886	0.598	7.49E-04	4.91E-02
KRTAP4-2	-5.402	1.56E-09	7.92E-06		NELL2	-0.870	5.28E-04	4.01E-02		C10orf90	0.601	9.39E-05	1.50E-02
KRTAP8-1	-5.376	4.57E-09	1.04E-05		PRKXP1	-0.863	1.16E-05	3.64E-03		LINC01198	0.606	2.50E-04	2.65E-02
KRTAP1-1	-5.325	2.74E-09	8.71E-06		PALLD	-0.862	2.11E-05	5.74E-03		MMP27	0.606	6.80E-04	4.59E-02
KRTAP1-3	-5.302	3.58E-09	1.01E-05		FZD3	-0.853	1.41E-04	1.94E-02		ADD3-AS1	0.608	1.61E-04	2.12E-02
KRTAP3-2	-5.292	2.79E-09	8.71E-06		CYFIP2	-0.820	1.48E-04	2.02E-02		BST1	0.625	3.29E-05	7.75E-03
KRTAP2-1	-5.290	4.01E-09	1.01E-05		FLRT3	-0.791	1.41E-06	7.73E-04		TLR1	0.640	6.11E-04	4.31E-02
KRTAP9-9	-5.263	3.68E-09	1.01E-05		UBE2H	-0.783	3.13E-07	2.42E-04		NRG4	0.644	2.09E-04	2.38E-02
KRTAP2-2	-5.163	5.23E-09	1.10E-05		SULF2	-0.779	8.53E-07	5.02E-04		ZFPM2	0.648	9.29E-05	1.49E-02
KRTAP4-7	-5.122	4.23E-09	1.01E-05		TCP11L2	-0.772	4.70E-06	2.07E-03		STAT4	0.651	5.89E-04	4.24E-02
KRTAP9-3	-5.115	9.15E-10	7.08E-06		WFDC1	-0.771	6.89E-04	4.63E-02		PRKCB	0.653	2.75E-04	2.82E-02
KRTAP4-5	-5.090	6.52E-09	1.23E-05		SLC39A10	-0.759	5.76E-07	3.66E-04		PDGFRL	0.671	8.85E-05	1.46E-02
KRTAP3-1	-4.982	1.53E-08	2.40E-05		CCNA1	-0.746	2.12E-04	2.39E-02		HS3ST4	0.678	1.61E-05	4.60E-03
KRTAP9-2	-4.956	7.23E-09	1.32E-05		ARL4C	-0.741	7.99E-06	2.93E-03		ZNF436-AS1	0.683	1.60E-05	4.60E-03
KRTAP4-3	-4.955	2.46E-09	8.71E-06		MTCL1	-0.734	4.38E-04	3.58E-02		MLXIPL	0.687	5.42E-04	4.04E-02
KRT34	-4.933	2.50E-09	8.71E-06		S100A2	-0.734	3.11E-04	3.01E-02		SLITRK4	0.692	2.96E-04	2.93E-02
KRTAP19-1	-4.911	2.09E-10	6.54E-06		PLEKHG1	-0.721	4.66E-06	2.07E-03		NEFL	0.693	6.17E-06	2.54E-03
KRT33B	-4.836	3.32E-08	4.42E-05		TNC	-0.706	7.58E-06	2.86E-03		TMEM200C	0.693	2.08E-04	2.38E-02
KRTAP4-9	-4.731	1.76E-08	2.60E-05		CXCR2	-0.704	5.47E-05	1.04E-02		RBM26-AS1	0.698	8.41E-05	1.42E-02
KRTAP3-3	-4.730	8.68E-08	9.16E-05		NBPF14	-0.703	3.06E-04	2.98E-02		MYLK4	0.710	1.79E-04	2.21E-02
KRTAP4-11	-4.511	6.03E-10	7.08E-06		CYP24A1	-0.700	1.93E-04	2.28E-02		RGN	0.710	2.28E-04	2.50E-02
KRTAP11-1	-4.440	8.71E-08	9.16E-05		GALNT6	-0.698	1.81E-04	2.22E-02		GALNT12	0.711	6.45E-05	1.18E-02
KRT33A	-4.287	3.00E-08	4.29E-05		AMBN	-0.696	3.27E-07	2.48E-04		TRAM1L1	0.711	5.78E-05	1.09E-02
KRTAP19-3	-4.267	1.04E-09	7.08E-06		SOSTDC1	-0.691	1.72E-06	9.13E-04		RCAN2	0.715	3.81E-05	8.58E-03
KRTAP4-8	-4.149	1.46E-08	2.35E-05		KRT74	-0.689	2.13E-05	5.77E-03		ENPP5	0.723	6.97E-04	4.65E-02
GPRC5D	-4.036	5.81E-09	1.18E-05		TGIF1	-0.668	9.97E-05	1.54E-02		NPR3	0.724	2.97E-04	2.93E-02
KRTAP10-11	-3.932	1.29E-08	2.28E-05		ADAMTS1	-0.665	1.54E-04	2.05E-02		BCHE	0.730	2.30E-04	2.51E-02
KRTAP7-1	-3.926	8.55E-08	9.16E-05		MLLT11	-0.661	1.02E-06	5.82E-04		SLC35F1	0.746	2.11E-04	2.39E-02
KRT86	-3.799	1.72E-08	2.60E-05		SNORA28	-0.660	6.28E-04	4.37E-02		HSPA12A	0.746	3.34E-06	1.59E-03
KRTAP17-1	-3.781	3.11E-08	4.29E-05		VSNL1	-0.642	3.38E-07	2.48E-04		PRG2	0.750	4.95E-04	3.84E-02
KRT81	-3.720	3.14E-08	4.29E-05		SERPINB13	-0.636	5.34E-05	1.03E-02		IGSF10	0.751	1.99E-04	2.32E-02
KRT35	-3.627	1.23E-05	3.75E-03		BNC1	-0.628	1.46E-05	4.30E-03		CACNA1D	0.767	1.74E-04	2.17E-02
KRT83	-3.591	6.70E-08	7.79E-05		F11R	-0.612	1.99E-04	2.32E-02		YBX2	0.785	2.81E-04	2.86E-02
LY6G6D	-3.551	1.19E-07	1.19E-04		CCN2	-0.612	3.23E-04	3.04E-02		EPDR1	0.789	2.54E-04	2.67E-02
KRTAP5-8	-3.473	1.43E-08	2.35E-05		FAM83A	-0.608	3.98E-06	1.84E-03		CLIC6	0.798	4.83E-04	3.78E-02
KRT85	-3.321	4.04E-06	1.85E-03		DLGAP5	-0.607	3.57E-04	3.19E-02		PLEKHG5	0.799	2.79E-04	2.85E-02
CBLN2	-3.298	6.82E-06	2.70E-03		ANOS1	-0.606	4.44E-04	3.59E-02		UNC80	0.802	1.01E-04	1.54E-02
KRT38	-3.206	7.24E-08	8.24E-05		HSPA2	-0.603	7.71E-05	1.35E-02		RERGL	0.811	5.13E-04	3.94E-02
KRT31	-3.176	4.47E-07	2.97E-04		ZFR	-0.602	1.64E-04	2.13E-02		CCBE1	0.842	1.07E-04	1.59E-02
DSG4	-3.035	4.18E-06	1.89E-03		GJB6	-0.601	1.86E-07	1.66E-04		SGCG	0.865	3.35E-04	3.11E-02
KRTAP13-1	-3.018	5.40E-07	3.47E-04		SOX9	-0.598	2.91E-04	2.91E-02		MFSD4A	0.873	1.63E-04	2.13E-02
KRT82	-2.983	1.26E-07	1.23E-04		KRT36	-0.597	1.51E-04	2.04E-02		SERPINB9P1	0.903	5.25E-05	1.03E-02
KRT32	-2.870	2.49E-07	2.07E-04		FZD6	-0.597	1.45E-06	7.85E-04		GRB14	0.930	3.42E-04	3.13E-02
PRR9	-2.842	7.94E-06	2.93E-03		SLC35F3	-0.596	6.02E-05	1.12E-02		KLB	1.023	7.51E-04	4.91E-02
CHAC1	-2.776	6.35E-08	7.54E-05		NRARP	-0.593	3.94E-04	3.39E-02		C14orf39	1.031	3.56E-04	3.18E-02
ANGPTL7	-2.670	7.46E-07	4.44E-04		C12orf75	-0.592	6.75E-04	4.57E-02		CLCNKB	1.220	4.32E-04	3.55E-02
KRT27	-2.510	1.60E-05	4.60E-03		OVOL1	-0.592	2.89E-05	7.05E-03		PCSK2	1.277	4.51E-08	5.61E-05
PADI3	-2.385	6.73E-07	4.13E-04		RCN2	-0.591	7.17E-04	4.75E-02		FAM3B	1.305	2.37E-07	2.00E-04
TCHH	-2.369	2.70E-05	6.88E-03		ARL15	-0.591	4.18E-05	9.11E-03					
S100A3	-2.208	9.79E-08	1.01E-04		PGBD5	-0.590	4.42E-04	3.59E-02					
KRTAP10-12	-2.172	4.73E-07	3.08E-04		MARCKSL1	-0.584	3.32E-04	3.09E-02					
COMP	-2.068	3.49E-06	1.64E-03		ME3	0.581	4.16E-04	3.50E-02					
KRT71	-2.067	1.55E-04	2.06E-02		MAN1C1	0.582	2.85E-04	2.88E-02					
LYG2	-1.980	1.98E-07	1.71E-04		FMOD	0.586	2.37E-04	2.56E-02					
KRT75	-1.898	2.88E-07	2.28E-04		CHDH	0.591	3.25E-04	3.06E-02					
PSORS1C2	-1.846	3.56E-07	2.49E-04		IL15	0.595	3.70E-04	3.26E-02					
SHISA2	-1.792	3.30E-06	1.58E-03		RNF150	0.597	6.75E-04	4.57E-02					
PIRT	-1.737	5.72E-08	6.96E-05		LINC00886	0.598	7.49E-04	4.91E-02					
SERPINA1	-1.735	7.31E-06	2.79E-03		C10orf90	0.601	9.39E-05	1.50E-02					
SERPINA3	-1.623	1.22E-05	3.75E-03		LINC01198	0.606	2.50E-04	2.65E-02					
KRTAP5-9	-1.580	6.00E-06	2.50E-03		MMP27	0.606	6.80E-04	4.59E-02					
CST1	-1.570	1.06E-05	3.42E-03		ADD3-AS1	0.608	1.61E-04	2.12E-02					
WNK4	-1.537	1.02E-05	3.41E-03		BST1	0.625	3.29E-05	7.75E-03					
ATP8A2	-1.530	1.05E-05	3.42E-03		TLR1	0.640	6.11E-04	4.31E-02					
KRT16	-1.468	2.24E-06	1.12E-03		NRG4	0.644	2.09E-04	2.38E-02					
RNF182	-1.443	4.87E-05	9.93E-03		ZFPM2	0.648	9.29E-05	1.49E-02					
SLC27A6	-1.427	4.10E-07	2.79E-04		STAT4	0.651	5.89E-04	4.24E-02					
HOXC13	-1.424	4.38E-08	5.56E-05		PRKCB	0.653	2.75E-04	2.82E-02					
LYPD6	-1.396	1.16E-06	6.41E-04		PDGFRL	0.671	8.85E-05	1.46E-02					
PARM1	-1.394	4.23E-08	5.50E-05		HS3ST4	0.678	1.61E-05	4.60E-03					
SMTNL2	-1.386	1.47E-05	4.33E-03		ZNF436-AS1	0.683	1.60E-05	4.60E-03					
CREB5	-1.355	1.37E-08	2.33E-05		MLXIPL	0.687	5.42E-04	4.04E-02					
SLN	-1.345	4.80E-06	2.10E-03		SLITRK4	0.692	2.96E-04	2.93E-02					
COL11A1	-1.301	2.04E-04	2.36E-02		NEFL	0.693	6.17E-06	2.54E-03					
EGR1	-1.266	4.76E-05	9.79E-03		TMEM200C	0.693	2.08E-04	2.38E-02					
CYRIA	-1.265	1.03E-05	3.41E-03		RBM26-AS1	0.698	8.41E-05	1.42E-02					
KRT72	-1.248	3.83E-04	3.31E-02		MYLK4	0.710	1.79E-04	2.21E-02					
KLK6	-1.175	3.10E-04	3.01E-02		RGN	0.710	2.28E-04	2.50E-02					
SLC7A8	-1.158	2.20E-06	1.12E-03		GALNT12	0.711	6.45E-05	1.18E-02					
BMP2	-1.147	8.60E-06	3.06E-03		TRAM1L1	0.711	5.78E-05	1.09E-02					
TENM2	-1.139	3.56E-07	2.49E-04		RCAN2	0.715	3.81E-05	8.58E-03					
CHST2	-1.129	2.59E-05	6.61E-03		ENPP5	0.723	6.97E-04	4.65E-02					
PKP1	-1.102	2.58E-05	6.61E-03		NPR3	0.724	2.97E-04	2.93E-02					
CYP27B1	-1.096	2.10E-04	2.39E-02		BCHE	0.730	2.30E-04	2.51E-02					
HAPLN1	-1.074	1.48E-04	2.03E-02		SLC35F1	0.746	2.11E-04	2.39E-02					
SAMD5	-1.071	7.08E-07	4.26E-04		HSPA12A	0.746	3.34E-06	1.59E-03					
CAPN12	-1.070	4.46E-05	9.52E-03		PRG2	0.750	4.95E-04	3.84E-02					
KRT40	-1.066	4.96E-06	2.14E-03		IGSF10	0.751	1.99E-04	2.32E-02					
SLC16A9	-1.057	3.74E-06	1.75E-03		CACNA1D	0.767	1.74E-04	2.17E-02					
PTP4A1	-1.055	9.17E-05	1.48E-02		YBX2	0.785	2.81E-04	2.86E-02					
FOXE1	-1.050	1.63E-04	2.13E-02		EPDR1	0.789	2.54E-04	2.67E-02					
AREG	-1.033	1.19E-04	1.70E-02		CLIC6	0.798	4.83E-04	3.78E-02					
EMP1	-1.000	4.43E-04	3.59E-02		PLEKHG5	0.799	2.79E-04	2.85E-02					
KRT6B	-0.992	1.51E-04	2.04E-02		UNC80	0.802	1.01E-04	1.54E-02					
OXCT2	-0.978	5.94E-06	2.50E-03		RERGL	0.811	5.13E-04	3.94E-02					
FNDC1	-0.970	2.78E-04	2.85E-02		CCBE1	0.842	1.07E-04	1.59E-02					
TRIM9	-0.946	6.74E-04	4.57E-02		SGCG	0.865	3.35E-04	3.11E-02					
KLB	1.023	7.51E-04	4.91E-02		MFSD4A	0.873	1.63E-04	2.13E-02					
C14orf39	1.031	3.56E-04	3.18E-02		SERPINB9P1	0.903	5.25E-05	1.03E-02					
CLCNKB	1.220	4.32E-04	3.55E-02		GRB14	0.930	3.42E-04	3.13E-02					
PCSK2	1.277	4.51E-08	5.61E-05		FAM3B	1.305	2.37E-07	2.00E-04					

Supplementary Methods

Materials and Methods

Datasets

Our analysis integrated transcriptomic data from multiple sources, including Pfizer's phase II study (ALLEGRO) and publicly available datasets comprising lesional and non-lesional scalp biopsies from patients with alopecia areata (AA) (GSE148346[1], GSE68801[2], GSE45512[3], GSE111061[4]) and normal controls (GSE68801, GSE45512, GSE111061) (Supplementary Table 2). Transcriptomic studies using whole scalp tissue from controlled clinical trials are rare. The selected datasets represent all such datasets available to Pfizer at the time of analysis. Among these, GSE148346 and GSE68801 had the largest sample sizes. GSE148346 was generated by Pfizer,[1] while the others originated from independent academic studies.[2–4] All datasets used the Affymetrix HGU133plus2.0 platform, ensuring consistency across platforms for the AA-focused analyses.

Serum samples were centrifuged and stored at −80°C. Protein measurements were performed using the Olink Proseek Multiplex platform (Olink Bioscience, Uppsala, Sweden), using inflammation, neuroinflammatory, and cardiovascular panels (CVD II and III), as previously described.[1]

Preprocessing and Gene Expression Data

Transcriptomic profiling from GSE148346 (Pfizer) was conducted using the Affymetrix HGU133plus2.0 array. Published studies (GSE68801, GSE45512, GSE111061)[2–4] also used this platform. Preprocessing and normalization were performed uniformly to allow comparison across studies. Differential gene expression between AA lesional and non-lesional scalps, as well as between AAP vs normal, and AT/AU vs normal, was performed using the LIMMA package from Bioconductor, which applies linear modeling with empirical Bayes approach and moderated t-statistics.[5]

Protein Expression Analysis

Protein expression differences between AAP, AT/AU, and normal controls were assessed using a linear model (lm function in R, v4.3.1).[7] Group (Normal, AAP, AT/AU) was modeled as a main effect. P-values were adjusted for multiple comparisons using the Benjamini-Hochberg (BH) procedure.[8]

Pathway Enrichment Analysis

Pathway analysis was conducted using the fgsea algorithm, which implements fast pre-ranked gene set enrichment analysis.[9] T-statistics from the linear model were used to rank genes. Pathway gene sets were obtained from MSigDB HALLMARK, REACTOME, and BIOCARTA/KEGG collections. P-values were adjusted using the BH method, with an FDR ≤ 0.05 considered significant.

Normalized enrichment scores (NES) were used to indicate the magnitude and direction of pathway activity, accounting for variation in gene set size and rank position.

Meta-analysis

An inverse-variance weighted meta-analysis was performed across studies with the contrasts of interest (see Supplementary Table 2) to estimate combined differences for each gene. Pooled p-values and fold changes were calculated across datasets, and BH correction was applied.[8] Genes with consistent direction and minimal heterogeneity were defined as core transcriptomic signatures for AAP or AT/AU. QQ plots of heterogeneity p-values indicated low inter-study variability overall, though some heterogeneity was observed within subsets of genes.

Statistical Software

All statistical analyses were conducted in R (v4.3.1)[7] using Bioconductor packages (www.bioconductor.org). Microarray intensities were normalized using GCRMA.[10]

References

1. Guttman-Yassky E, Pavel AB, Diaz A, Cardinale I, Fernandez X, Morselli D, et al. Ritlecitinib and brepocitinib demonstrate significant improvement in scalp alopecia areata biomarkers. J Allergy Clin Immunol 2022;149:1318-1328.
2. Jabbari A, Cerise JE, Chen JC, Mackay-Wiggan J, Duvic M, Price V, et al. Molecular signatures define alopecia areata subtypes and transcriptional biomarkers. EBioMedicine 2016;7:240–7.
3. Jabbari A, Nguyen N, Cerise JE, Ulerio G, de Jong A, Garza LA, et al. An open-label pilot study to evaluate the efficacy of tofacitinib in moderate to severe patch-type alopecia areata, totalis, and universalis. J Invest Dermatol 2018;138:1539–45.
4. Xing L, Dai Z, Jabbari A, Cerise JE, Higgins CA, Gong W, et al. Alopecia areata is driven by cytotoxic T lymphocytes and is reversed by JAK inhibition. Nat Med 2014;20:1043–9.
5. Smyth GK. Linear models and empirical Bayes methods for assessing differential expression in microarray experiments. Stat Appl Genet Mol Biol 2004;3:Article 3.
6. Wu Z, Irizarry RA, Gentleman R, Martinez-Murillo F, Spencer F. A model-based background adjustment for oligonucleotide expression arrays. J Am Stat Assoc 2004;99:909–17.
7. Korotkevich G, Sukhov V, Budin N, Shpak B, Artyomov MN, Sergushichev A. Fast gene set enrichment analysis. bioRxiv 2019.
8. Benjamini, Y.; Hochberg, Y. Controlling the false discovery rate: A practical and powerful approach to multiple testing. *J. R. Stat. Soc. Ser. B Methodol.* **1995**, *57*, 289–300.
9. Glickman JW, Dubin C, Renert-Yuval Y, et al. Cross-sectional study of blood biomarkers of patients with moderate to severe alopecia areata reveals systemic immune and cardiovascular biomarker dysregulation. J Am Acad Dermatol. Feb 2021;84(2):370-380.

10. Lind L, Ärnlöv J, Lindahl B, Siegbahn A, Sundström J, Ingelsson E. Use of a proximity extension assay proteomics chip to discover new biomarkers for human atherosclerosis. Atherosclerosis. Sep 2015;242(1):205-10.
